# Progress in tension-relieving suturing surgery: revolutionary surgical techniques and patient prognosis evaluation methods

**DOI:** 10.3389/fsurg.2025.1587582

**Published:** 2025-05-13

**Authors:** Maolong Ge, Weifeng Zheng, Ping Yao, Lin Gao, Xinyang Ge, Qixin Zhang, Xiaolin Wang, Chuangju Guo

**Affiliations:** ^1^ College of Mathematical Medicine, Zhejiang Normal University, Jinhua, China; ^2^ Department of Cosmetic Dermatology, Hangzhou Plastic Surgery Hospital, Hangzhou, Zhejiang, China; ^3^ Department of Plastic Surgery, Hangzhou Plastic Surgery Hospital, Hangzhou, Zhejiang, China; ^4^ Research and Development Department, Zhejiang Demetics Medical Technology Co., Ltd., Hangzhou, China

**Keywords:** tension-relieving suture surgery, surgical innovation, tension-related complications, wound healing, surgical prognosis

## Abstract

During the repair of skin and soft tissue wounds, excessive tension frequently leads to delayed healing, hypertrophic scarring, and tissue inflammation. Consequently, reducing skin or soft tissue tension has emerged as a crucial measure to facilitate wound healing. Tension-relieving suture procedures achieve this by adjusting incision design or employing specialized suturing techniques to release or diminish the tension at the wound margins, thereby improving local blood circulation and cellular signaling, promoting wound closure, and reducing scar formation. The super-tension-relieving suture procedure, an innovative advancement in tension-relieving technology, utilizes slowly absorbable barbed sutures to extend the duration of tension relief. This method is particularly advantageous in cases involving large excision areas and high tension, as it offers significant benefits over traditional techniques in terms of suturing approach, tension dispersion, and local stress regulation, thereby more effectively preventing hypertrophic scars. Moreover, preliminary studies suggest that it exerts a positive impact on the management of keloids. This review comprehensively examines the evolution, design principles, and clinical applications—with evidence-based outcomes—of tension-relieving suture techniques across multiple disciplines, including plastic surgery, cardiovascular surgery, neurosurgery, and orthopedics. By integrating a wealth of literature and clinical data, the paper aims to elucidate the significant benefits and future prospects of tension-relieving strategies in enhancing wound repair quality, lowering scar risk, and advancing surgical innovation.

## 1 Introduction

Surgical intervention for wound healing is an indispensable part of modern medical practice, ranging from routine reconstructive procedures to complex reconstructive surgeries. However, tension-related complications, such as wound dehiscence, tissue necrosis, and impaired healing, present significant challenges to the success of surgeries and the prognosis of patient wounds (1, 2). In the field of plastic surgery, studies have shown that there are significant differences in the mechanical properties between normal skin and scar tissue (3, 4). For example, under identical loading conditions, the stiffness of forearm scar tissue decreases from 3.4 N/mm in grade 5 scars to 0.5 N/mm in grade 1 scars, whereas normal skin exhibits a stiffness of approximately 0.042 N/mm (5). Given the differences in these mechanical properties and their role in pathological scar formation, various tension-relieving suture techniques have been progressively explored in clinical practice to modulate the mechanical forces at the wound edges, thereby improving the healing process and reducing the risk of abnormal scar formation. Pathological scars (PS), including keloids and hypertrophic scars (HS), are formed as a result of abnormal wound healing processes. Mechanical forces at the injured skin, such as tension, shear, and compressive forces, are the primary triggering factors for the growth and formation of PS (6). Tension-relieving suture surgery plays a critical role in intervening in wound healing and scar appearance. Different suturing techniques directly influence healing outcomes and the final scar morphology by adjusting the method of wound closure and tension distribution. Common tension-relieving suture techniques include interrupted sutures, continuous sutures, mattress sutures, and buried sutures. The final cosmetic outcome of surgical wounds depends on several factors, including skin tension, the extent of injury, and the presence of infection, with skin tension being a significant determinant of scar formation and progression. The use of appropriate tension-relieving methods can reduce the tension at the wound edges, thereby inhibiting hypertrophic scar formation (7). At the same time, this technique should not be time-consuming or difficult to implement (8). Buried suturing techniques meet these standards, achieving minimal postoperative care, no need for suture removal, and no visible suture marks (8). In thoracic surgery, similar challenges are faced with wounds after heart and lung surgeries, especially in sternotomy wound infection repair, where excessive suturing tension may lead to abnormal protrusion or deformation of the chest wall (9). In recent years, advancements in tension-relieving suture surgery have sparked widespread interest in the medical field and have yielded positive academic and prognostic evaluations. This review aims to explore the development, concepts, technical characteristics, clinical practices, and potential impacts of tension-relieving suture surgery on surgical outcomes and patient recovery.

## 2 Methods

This study conducted a systematic review of the development, conceptual framework, clinical applications, and outcomes of tension-relieving suture surgery by searching the PubMed, Google Scholar, Web of Science and Baidu Scholar databases. The search period was set from 1897 to 2024 to comprehensively encompass both the early explorations and the latest advancements in the field. The primary search keywords included “Tension-relieving suture surgery,” “Surgical innovation,” and “Tension-related complications.” Inclusion criteria comprised original research articles, systematic reviews, retrospective analyses, technical reports, and historical literature reviews published in either English or Chinese. Additionally, the analysis of historical literature focused on delineating the origins of the tension-relief concept, the key milestones in technological innovation, and its adaptive development in modern surgery, thereby systematically elucidating the complete trajectory of tension-relieving suture surgery from traditional techniques to contemporary optimizations and its clinical significance.

## 3 Development of tension-relieving suture surgery

**Figure 1:**
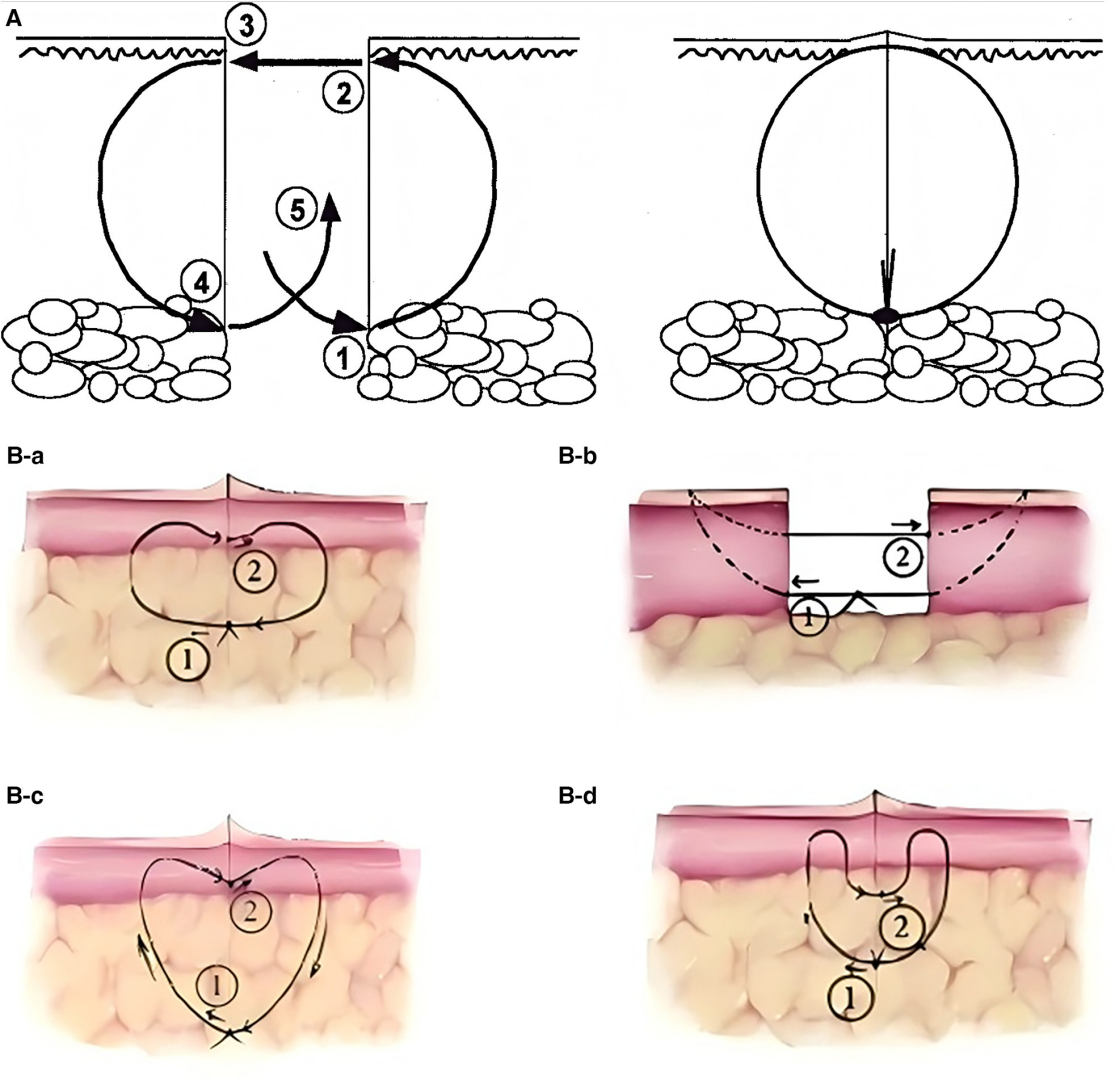
**(A)** Buried dermal suture: before suturing (left), after suturing (right). **(B)** Vertical mattress suture and its modified techniques: (a) BVMS. (b) MBVMS. (c) WEMVMS. (d) SBDS. Reproduced with permission from Acta Derm Venereol (16) and Chinese Journal of Reparative and Reconstructive Surgery (17).

Since the concept of buried suture techniques was first introduced by Halsted in the late 19th century (10), various modified approaches have been progressively developed. The core objectives of these techniques remain consistent: to reduce infection, promote wound healing, and optimize scar appearance (11) (Table 1). These buried sutures are entirely located beneath the skin surface, thereby avoiding contact with external contaminants (Figure 1A). In 1989, Zitelli et al. (12) introduced the Buried Vertical Mattress Suture (BVMS) (Figure 1B-a), which achieves deep dermal approximation and knot placement in the subcutaneous fat layer. This approach effectively reduces scar formation but is less suitable for thin-skinned areas such as the periorbital region. To overcome the limitations of conventional BVMS, a series of improved techniques have since emerged. In 1994, Sadick et al. (13) proposed the Modified Buried Vertical Mattress Suture (MBVMS) (Figure 1B-b), which enhances wound edge approximation by employing *in situ* backstitching and symmetric needle pathways. Building on this, Zhang et al. (14) introduced in 2009 the Wedge-Shaped Excision Combined with Modified BVMS (WEMVMS) (Figure 1B-c). This method not only improves epidermal eversion but also increases tissue anchoring by extending the intradermal suture path. Additionally, the knot is relocated to the dermal-fat junction to reduce local irritation. Due to the characteristic shape of the suture pattern, this technique is also referred to as the “heart-shaped suture.” In 2010, Daneshpazhooh et al. (15). proposed the Setback Buried Dermal Suture (SBDS) (Figure 1B-d), in which the needle entry and exit points are positioned 2–3 mm away from the wound edge. This modification minimizes suture-induced trauma at the wound margin while significantly enhancing wound edge eversion and tension-relieving effects.

**Table 1:** Representative tension-relieving buried suture techniques and their Key features.

Technique	Year	Developer	Key features	Advantages	Limitations
BVMS	1989	Zitelli et al.	Vertical dermal trajectory with subcutaneous knot	Moderate eversion, no external marks	Not suitable for thin skin
MBVMS	1994	Sadick et al.	Needle re-entry at same site, superficial dermal exit	Enhanced control for thinner areas	More complex technique
WEMVMS	2009	Zhang et al.	Wedge excision, heart-shaped path	Strong dermal anchoring, reduced dermal irritation	Technically demanding
SBDS	2010	Daneshpazhooh et al.	2–3 mm setback needle entry	Eliminates wound-edge irritation	Demands precise needle positioning

**Figure 2:**
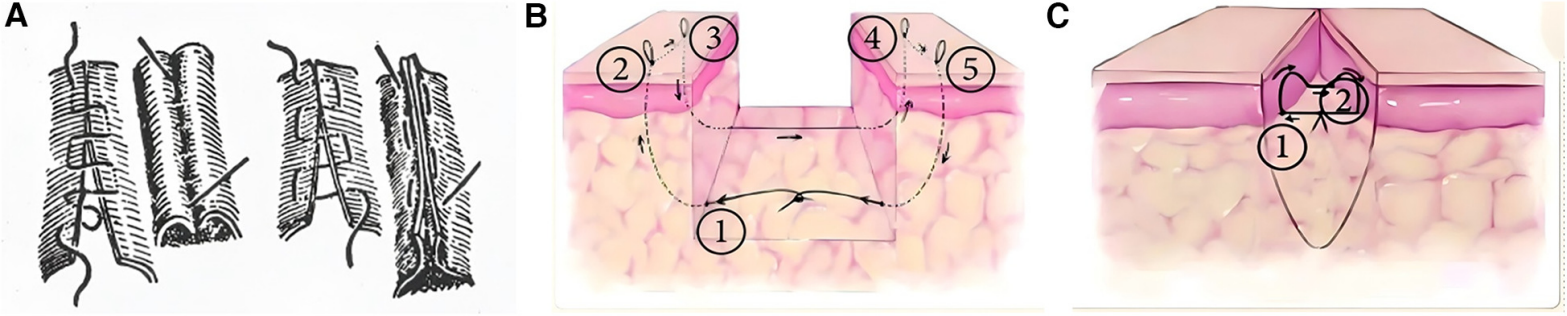
**(A)** Continuous horizontal mattress inversion suture. Buried horizontal mattress suture: **(B)** PBHMS. **(C)** FBHMS. Reproduced with permission from Chinese Journal of Reparative and Reconstructive Surgery (17).

**Figure 3:**
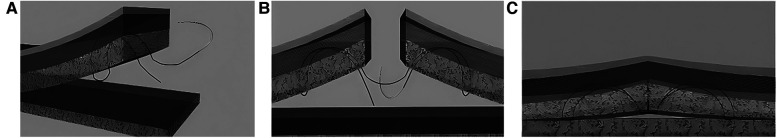
Schematic diagram of the modified FBHMS technique. **(A)** Suture path passing through one side of the incision; **(B)** Suture path passing through both sides of the incision; **(C)** Diagram after knotting. Reproduced with permission from Ann Plast Surg (21).

**Figure 4:**
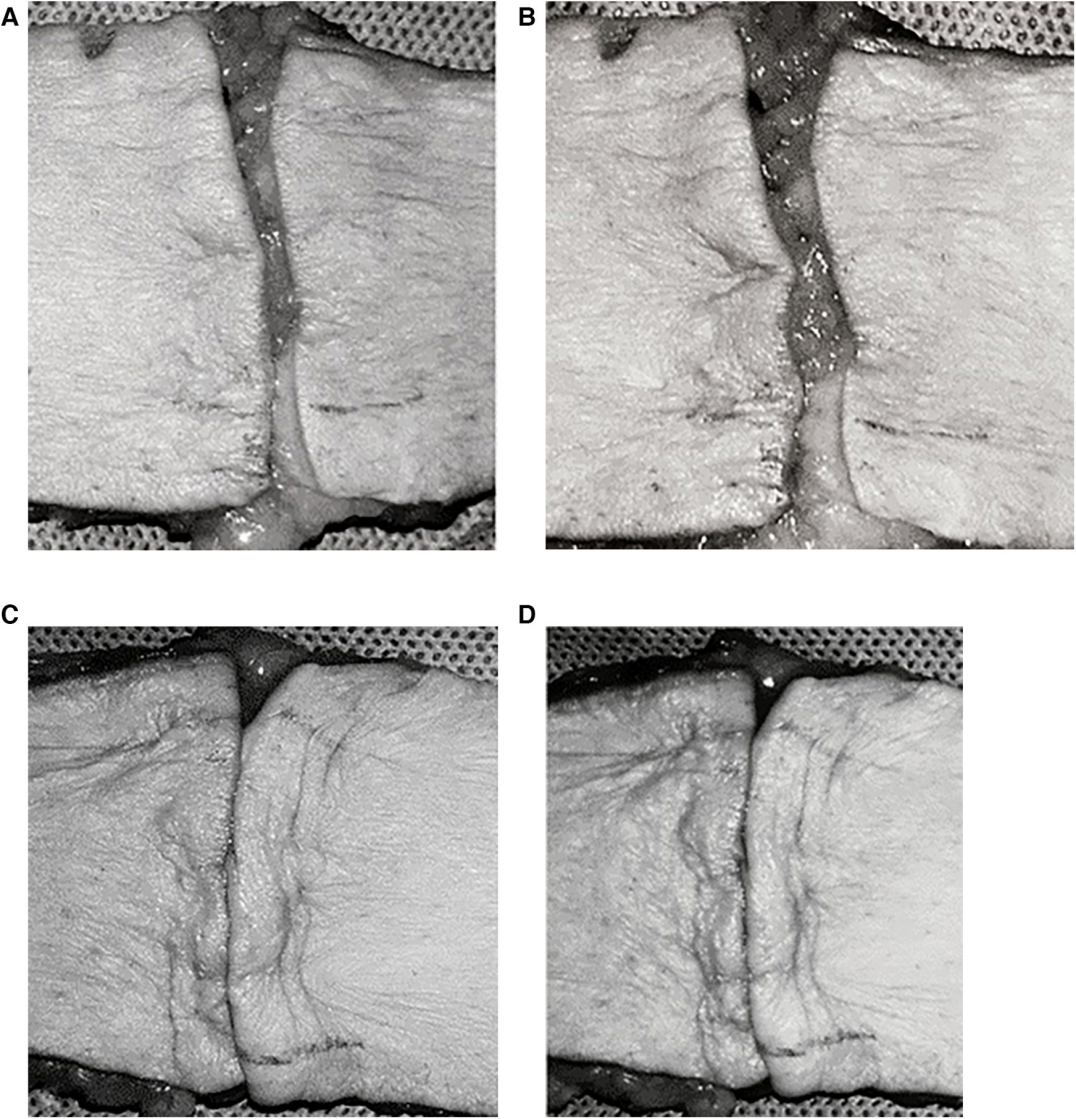
Control group: FBHMS experiment **(A,B)**; Experimental group: Modified FBHMS experiment **(C,D)**. After 100 cycles of periodic mechanical stress, the incision gap in the control group widened further, while the gap in the experimental group remained narrow. Reproduced with permission from Ann Plast Surg (21).

Horizontal Mattress Suture (HMS) (18) (Table 2), like the vertical mattress suture, is a classic technique that provides good tension relief, eversion or inversion (meaning the suture technique can cause the wound edges to evert or invert, allowing the edges to closely align, which promotes healing), hemostasis, and support for the incision. However, the exposed sutures often lead to noticeable scarring (Figure 2A). To enhance aesthetic outcomes, various buried horizontal mattress suture techniques have been developed in recent years. In 2004, See et al. (19) proposed the Percutaneous Buried Horizontal Mattress Suture (PBHMS) (Figure 2B), in which the needle penetrates the epidermis and is passed parallel within the dermis, thereby avoiding suture exposure. However, this technique may be associated with the formation of epidermoid cysts. Alam et al. (20) introduced the Fully Buried Horizontal Mattress Suture (FBHMS) (Figure 2C), with the suture embedded at the junction of the dermis and subcutaneous fat. While suitable for superficial incisions, this method provides limited tension-relieving effects in deeper or high-tension wounds. To address this limitation, Meng et al. (21) proposed a modified FBHMS technique (Figure 3), in which two arc-shaped suture loops are formed within the subcutaneous fat layer. This modification allows for greater tissue anchoring and significantly enhances tension relief.Experimental models using abdominal skin flaps post-abdominoplasty (Figure 4) demonstrated that this method effectively prevents wound dehiscence and maintains tight wound edge approximation even under cyclic mechanical stress, outperforming traditional suture techniques.

**Table 2:** Evolution of buried horizontal mattress sutures.

Technique	Year	Key features	Advantages	Limitation
HMS	2002	Classic exposed horizontal mattress suture	Tension relief, eversion, hemostasis	Scar formation due to exposed sutures
PBHMS	2004	Needle tunnels through dermis, avoids suture exposure	Cosmetic, tension relief	Technique complexity, risk of epidermoid cysts
FBHMS	2004	Entirely buried at dermal-fat junction	Good for superficial wounds	Limited for deep/high-tension wounds
Modified FBHMS	2017	Double arc loops in fat layer	Superior tension reduction, strong anchoring	Technically complex

Traditional tension-relieving suture techniques generally meet the requirements for wound edge approximation and eversion; however, the sutures remaining in the wound edge tissues continue to cause irritation, resulting in persistent tension. To overcome this, Zhang Yixin's team combined a special needle techniques and the characteristics of barbed sutures, proposed “Zhang's Super-Tension-Relieving Suture Surgery” (22). This technique anchors the dermal layers on both sides of the wound, redistributes the tension, and, by leveraging the special mechanical action of the barbed suture, disperses stress in high-tension areas to extend the duration of the tension-relieving effect. During the procedure, the method optimizes the way the suture passes through the skin, reducing irritation to the wound edge, shortening operating time, and improving recovery outcomes (with the tension-relieving effect lasting up to 26 weeks) as well as patient satisfaction (with an average Vancouver Scar Scale score of 2.1). Notably, super tension-relieving techniques are not only used to reduce the risk of hypertrophic scars, but preliminary studies have also suggested that they may exert a positive impact on the management of keloids. However, due to the relatively large diameter of barbed sutures, there is a potential risk of pigmentation and scar formation at the skin penetration points.

## 4 Clinical applications of tension-relieving suture surgery

Tension-relieving suture surgery has been widely applied across multiple surgical disciplines, including plastic surgery, general surgery, orthopedics, neurosurgery, and cardiovascular surgery. These techniques have demonstrated exceptional adaptability and effectiveness in addressing specific tension-related challenges in various anatomical regions and surgical procedures.

### 4.1 Clinical applications in plastic surgery

Tension-relieving suture surgery is extensively utilized in plastic surgery, particularly in facial scar management, breast reconstruction, and flap transplantation (23, 24). By reducing the mechanical traction at the wound edges to improve local microcirculation, and by appropriately distributing tension to minimize inflammation and tissue edema, scar expansion and postoperative complications are reduced. This tension regulation technique not only promotes wound healing by preventing dehiscence or irregular healing caused by excessive pulling, but also effectively inhibits scar hypertrophy, thereby significantly enhancing tissue apposition and functional recovery while improving aesthetic outcomes (25, 26).

**Figure 5:**
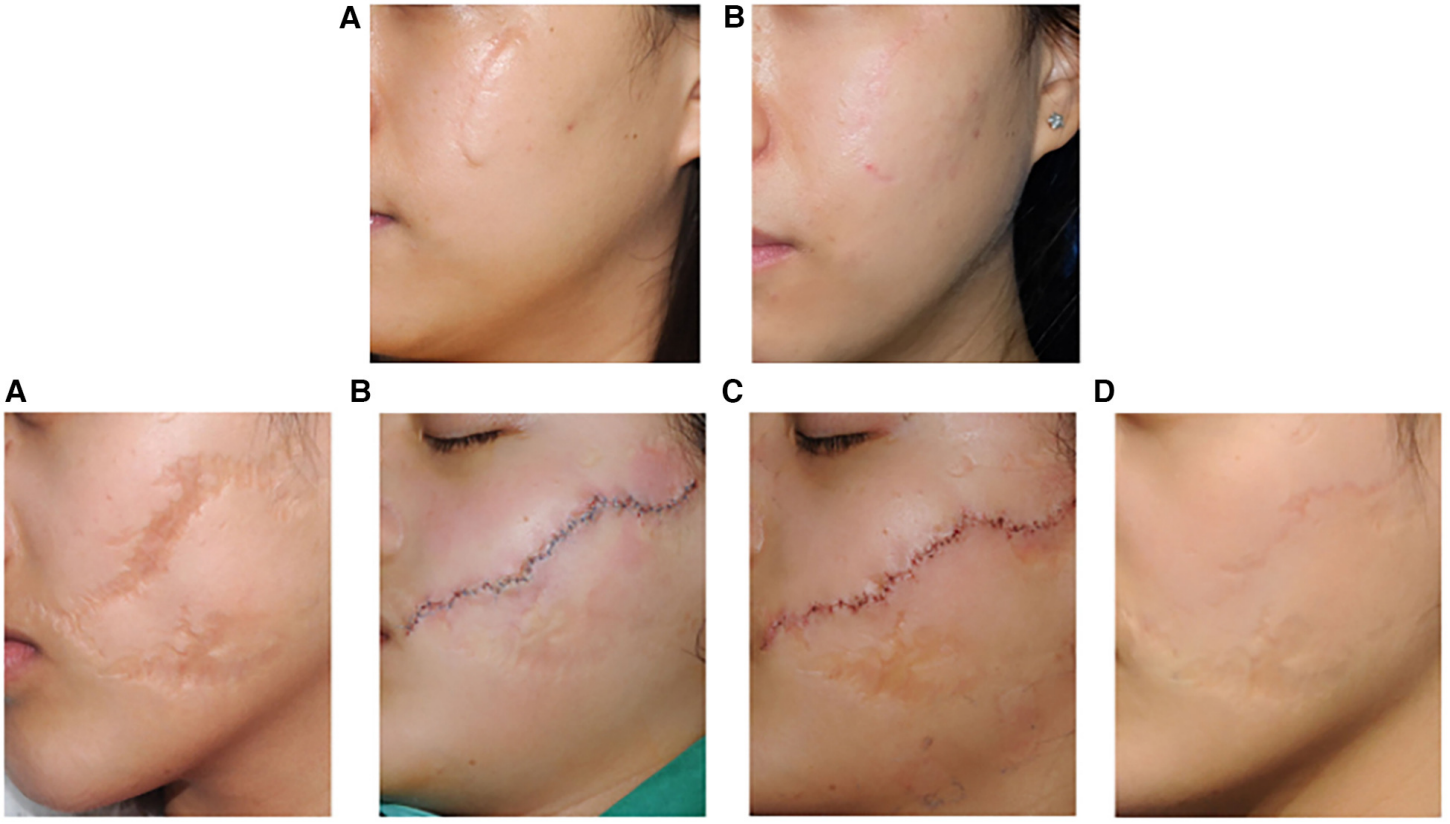
Representative clinical photographs of the first group (top): A 24-year-old female with a facial scar, treated with W-plasty incision suturing and CTR therapy. **(A)** Preoperative; **(B)** 12 months postoperatively; Preoperative VSS score: 5, postoperative VSS score: 1; Preoperative VAS score: 60, postoperative VAS score: 95. Representative clinical photographs of the second group (bottom): **(A)** 14- year-old female with a facial scar, treated only with W-plasty incision suturing. **(A)** Preoperative; **(B)** On the day of surgery; **(C)** One week postoperatively (after suture removal); **(D)** 12 months postoperatively; Preoperative VSS score: 7, postoperative VSS score: 4; Preoperative VAS score: 40, postoperative VAS score: 75. Reproduced with permission from Int Wound J (27).

Plastic surgery research has validated the effectiveness of tension-relieving suture surgery in promoting wound healing and improving cosmetic outcomes following complex reconstructive procedures. In a retrospective cohort study on the application of continuous tension-relieving suture surgery in facial scar treatment (27), 40 patients were divided into two groups. The first group underwent continuous tension-relieving suturing, while the second group received standard suturing without tension relief. Clinical results indicated a significant difference in scar appearance between the two groups, with tension-relieving sutures being closely associated with improved scar aesthetics and higher patient-reported satisfaction scores. The study utilized the Vancouver Scar Scale (VSS) (0–15), Visual Analog Scale (VAS) (0–100), and patient satisfaction scores to evaluate treatment outcomes. Table 3 summarizes the demographic characteristics of the participants. Representative clinical photographs are shown in Figure 5.

**Table 3:** Statistical data on patients and facial scars. Reproduced with permission from Int Wound J (27).

Patient	First group	Second group
Age	23.05 ± 10.9	21.5 ± 7.98
Sex		
Female	15	18
Male	5	2
Follow-up time (months)	12	12
Excision width (mm)	4.38 ± 2.53	4.78 ± 2.08
Scar length (cm)	9 ± 2.84	8.58 ± 2.24
Preoperative VAS Score	44.3 ± 10.23	48.5 ± 10.27
Postoperative VAS score	89.9 ± 3.9	76.65 ± 5.29
Preoperative VSS Score	8.85 ± 2.11	8.60 ± 1.66
Postoperative VSS score	1.7 ± 0.66	4.05 ± 1.10
Very satisfied	16 (80)	3 (15)
Satisfied	4 (20)	8 (40)
Less satisfied	0 (0)	7 (35)
Dissatisfied	0 (0)	2 (10)

In a clinical study investigating the application and efficacy of tension-relieving sutures in the repair of hypertrophic scars (28), 46 patients with hypertrophic scars underwent surgical treatment using tension-relieving suture surgery (patient demographics, including gender, age, and scar location, are shown in Table 4). The Patient and Observer Scar Assessment Scale (POSAS) and the Vancouver Scar Scale (VSS) were used to evaluate treatment outcomes before surgery and six months postoperatively. The results showed improved scar quality and reduced scar width compared to preoperative conditions (Table 4). POSAS and VSS scores, including individual parameters and total scores, demonstrated significant improvements postoperatively (Table 4).

**Table 4:** Patient demographics and preoperative and postoperative scar assessment parameters. Reproduced with permission from BMC Surgery (28).

Category	Indicator	Preoperative	Postoperative	Difference
Patient Information	Total Number	46		
	Gender (Male: Female)	17:29		
	Age (Mean ± Standard Deviation)	31.6 ± 14.2		
	Scar Location (Head & Neck/Torso/Limbs)	9/29/8		
Scar Parameters	Width (cm)	1.7 ± 0.6	0.7 ± 0.2	1.0 ± 0.4
	Length (cm)	4.4 ± 3.6	7.4 ± 3.7	3.0 ± 3.6
VSS Score	Color	1.8 ± 0.7	1.0 ± 0.2	0.8 ± 0.5
	Blood Vessel Distribution	1.8 ± 0.9	0.7 ± 0.3	1.1 ± 0.6
	Flexibility	1.9 ± 1.5	0.4 ± 0.3	1.5 ± 1.0
	Height	1.3 ± 0.9	0.2 ± 0.2	1.1 ± 0.5
	Total Score	6.6 ± 3.1	2.0 ± 0.7	4.6 ± 1.4
PSAS Score	Color	5.4 ± 2.6	3.0 ± 1.4	2.4 ± 1.8
	Hardness	4.7 ± 2.6	1.4 ± 0.4	3.3 ± 1.3
	Thickness	4.6 ± 2.8	1.5 ± 0.5	3.1 ± 1.7
	Irregularity	3.1 ± 2.2	2.3 ± 0.8	0.8 ± 0.6
	Pain Sensation	3.6 ± 2.0	1.7 ± 0.6	1.9 ± 1.1
	Itching Sensation	3.9 ± 2.2	1.7 ± 0.7	2.2 ± 1.2
	Overall Evaluation	5.8 ± 2.0	2.1 ± 0.5	3.7 ± 1.1
OSAS Score	Blood Vessel Distribution	7.4 ± 2.1	2.9 ± 1.3	4.2 ± 1.5
	Thickness	4.9 ± 2.2	1.3 ± 0.3	3.6 ± 1.2
	Color	5.2 ± 2.9	3.9 ± 1.8	1.3 ± 0.8
	Softness	5.1 ± 2.7	1.5 ± 1.0	3.6 ± 1.5
	Protrusion	2.6 ± 1.8	1.0 ± 0.6	1.6 ± 1.1
	Overall Evaluation	6.2 ± 2.1	1.7 ± 0.9	4.5 ± 1.9

A study evaluating the scar-minimizing effects of tension-relieving suture surgery (29) conducted a retrospective analysis of 64 patients treated with tension-relieving suture surgery. Assessment parameters included the Patient and Observer Scar Assessment Scale (POSAS), the Vancouver Scar Scale (VSS), and scar width, with evaluations conducted at 6 and 12 months postoperatively. Results at the 12-month follow-up showed that the POSAS and VSS scores in the conventional suturing group (32.58 ± 5.43, 3.58 ± 1.39) were significantly higher than those in the tension-relieving suture group (29.75 ± 3.56, 2.78 ± 1.17) (Table 5). The average scar width in the tension-relieving suture group (1.62 ± 0.36) was significantly smaller than that in the conventional suturing group (1.87 ± 0.42) (Table 5). This tension-relieving suture surgery effectively prevents prolonged localized eversion and other complications that are often associated with standard suturing methods. In summary, tension-relieving sutures have significant clinical value in treating hypertrophic scars and hold promising prospects for broader applications.

**Table 5:** Preoperative and postoperative scar width and assessment parameters. Reproduced with permission from Journal of Cosmetic Dermatology (29).

Category	Position	6 Months			12 Months		
		Normal suture	Tension-relieving suture	Difference	Normal suture	Tension-relieving suture	Difference
Scar Width (cm)	Face & Neck	1.17 ± 0.30	1.20 ± 0.33	0.308	1.44 ± 0.25	1.37 ± 0.34	0.760
	Chest & Back	1.70 ± 0.25	1.60 ± 0.31	1.035	2.14 ± 0.40	1.91 ± 0.24	2.033
	Limbs	1.56 ± 0.19	1.54 ± 0.25	0.270	1.96 ± 0.25	1.63 ± 0.30	3.585
	Other	1.84 ± 0.21	1.28 ± 0.37	3.723	2.23 ± 0.17	1.68 ± 0.35	3.998
	Total	1.42 ± 0.34	1.50 ± 0.36	1.292	1.87 ± 0.42	1.62 ± 0.36	3.616
POSAS	Face & Neck	26.24 ± 2.53	27.10 ± 2.21	1.173	28.00 ± 2.72	27.81 ± 3.22	0.206
	Chest & Back	36.12 ± 3.74	34.82 ± 2.30	1.221	39.00 ± 3.04	33.00 ± 3.04	5.754
	Limbs	32.61 ± 2.38	29.11 ± 2.25	4.534	34.22 ± 2.37	30.44 ± 2.04	5.129
	Other	24.38 ± 2.20	24.63 ± 1.69	0.255	27.25 ± 2.66	26.38 ± 1.41	0.817
	Total	30.42 ± 5.31	29.41 ± 4.15	1.199	32.58 ± 5.43	29.75 ± 3.56	3.487
VSS	Face & Neck	1.48 ± 0.98	2.05 ± 0.92	1.943	2.67 ± 1.06	2.48 ± 0.98	0.603
	Chest & Back	3.12 ± 0.78	3.35 ± 1.06	0.721	4.88 ± 0.86	3.35 ± 1.37	3.900
	Limbs	2.50 ± 1.25	2.28 ± 1.23	0.532	4.00 ± 0.91	2.83 ± 1.10	3.477
	Other	2.25 ± 0.89	1.75 ± 0.89	1.124	2.25 ± 1.16	2.25 ± 1.04	<0.001
	Total	2.30 ± 1.18	2.42 ± 1.18	0.575	3.58 ± 1.39	2.78 ± 1.17	3.523

In a retrospective study evaluating the use of the super-tension-relieving suture technique for the treatment of pathological scars (30), 120 patients underwent the procedure. Preoperatively, 80 patients were diagnosed with keloids and 40 with hypertrophic scars. Postoperatively, all patients achieved primary closure without any major complications. At the 12-month follow-up, compared with preoperative assessments, the POSAS scores decreased by 70%, the PSAS scores by 68%, and the OSAS scores by 72%, all of which were statistically significant. In another study employing the super-tension-relieving suture technique (31), 45 patients with chest keloids underwent the procedure. At the 12-month follow-up, the wounds in all 45 patients had essentially healed, and only one recurrence (2.2%) was observed over a two-year follow-up period.

### 4.2 Clinical applications in general surgery

**Figure 6:**
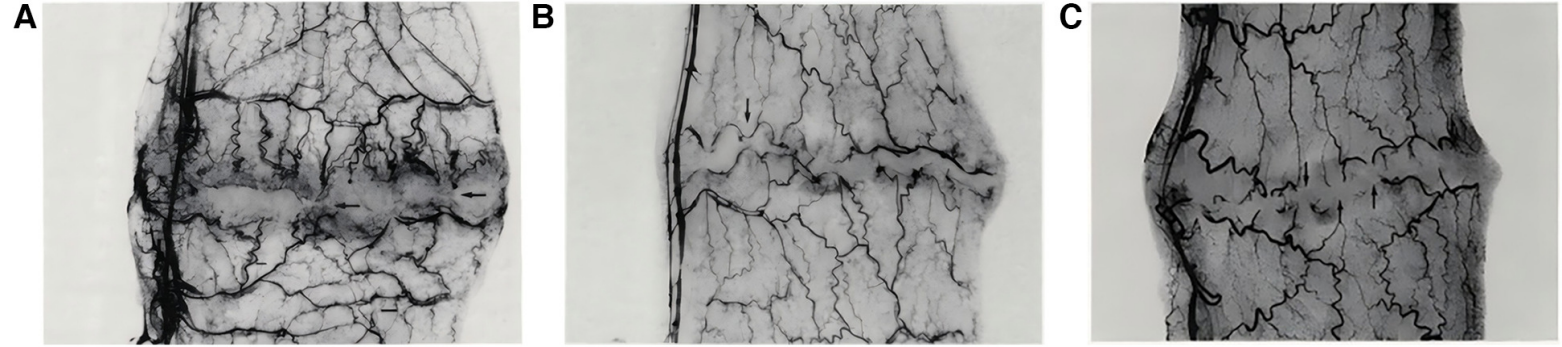
**(A)** Tension-relieving suturing allows new blood vessels to pass through the sutures around the anastomosis. **(B)** Moderate suture tension causes vessels to displace along the anastomosis. **(C)** High suture tension creates an avascular zone with vessel discoloration. Reproduced with permission from Am J Surg (34).

By reducing the mechanical traction at the wound edges to improve local microcirculation, and by appropriately distributing tension to minimize inflammation and tissue edema, scar expansion and postoperative complications are reduced. This tension regulation technique not only promotes wound healing by preventing dehiscence or irregular healing caused by excessive pulling, but also effectively inhibits scar hypertrophy, thereby significantly enhancing tissue apposition and functional recovery while improving aesthetic outcomes (32–34). In an experiment on the effect of suture tension on colon anastomosis healing (34), the study found that as suture tension increased, excessively high tension compressed the blood vessels in the anastomotic area, impairing local blood flow and leading to tissue ischemia. This lack of blood supply could affect the formation and healing of new tissue, increasing the risk of tissue necrosis. The experimental results showed that tension-relieving suturing allowed new blood vessels to grow through the sutures around the anastomosis (Figure 6A), moderate suture tension displaced the vessels but kept them patent (Figure 6B), while high tension formed an avascular zone around the anastomosis (Figure 6C).

### 4.3 Clinical applications in orthopedic surgery

**Figure 7:**
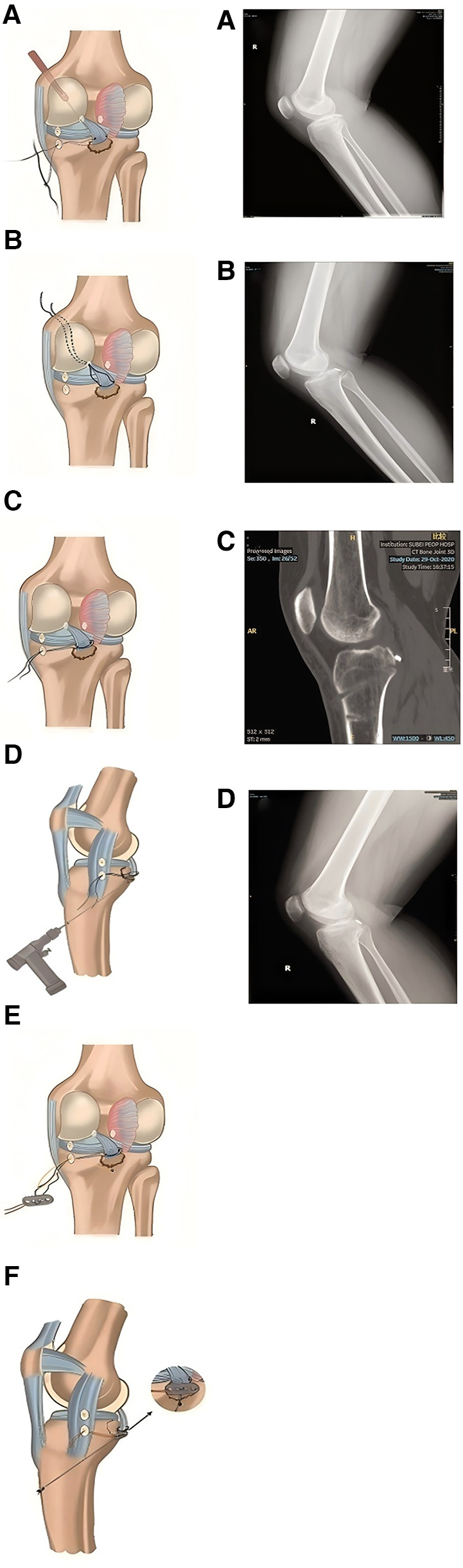
The doctor uses sutures, plates, and nails to fix the fracture site (left); CT scan of a 28- year-old female patient with a right posterior cruciate ligament avulsion fracture figure (right). **(A)** x-ray of the fracture; **(B)** x-ray taken on the first day after surgery; **(C)** CT image three months after surgery; **(D)** Bone fracture healing. Reproduced with permission from J Orthop Surg Res (35).

Orthopedic surgeons have adopted tension-relieving suture surgery because it optimizes soft tissue tension during joint reconstruction, tendon repair, and fracture fixation. In arthroscopic surgery, tension-relieving suturing allows for precise tension control and secure knotting, thereby improving the stability and longevity of ligament reconstruction and meniscus repair (as shown in the left part of Figure 7).

**Figure 8:**
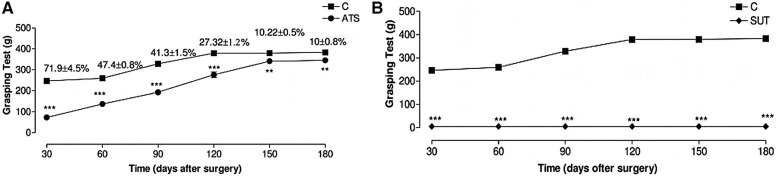
The effect of different surgical methods on the grip ability of Wistar rats. Results range from 30 days to 180 days. **(A)** Alleviated tension suture group. **(B)** Suture under tension group; Control group **(C)** did not undergo surgery, with each group representing the average score of 10 animals. Symbols indicate differences between groups. Reproduced with permission from Microsurgery (36).

In a postoperative follow-up study involving patients who underwent treatment for right posterior cruciate ligament avulsion fractures (35), tension-relieving suturing was found to better maintain the stability of the fracture site by optimizing tension and preventing suture relaxation, thereby promoting bone healing and shortening rehabilitation time. As shown in the right part of Figure 8, the x-ray taken on the first day after surgery shows satisfactory fracture relief. The CT scan performed three months post-surgery demonstrates good positioning and effect of the resorbable anchors. According to the evaluation of the Visual Analog Scale (VAS), knee range of motion, Lysholm score (used for functional assessment after knee joint injury), and the International Knee Documentation Committee (IKDC) pain assessment, the patient's knee mobility, pain relief, and overall knee function significantly improved (Table 6).

**Table 6:** Comparison of pain, knee range of motion, and function before and after surgery in patients. Reproduced with permission from J Orthop Surg Res (35).

Time point	VAS score	Knee extension	Knee flexion	Lysholm score	IKDC score
Preoperative	5.2 (4.0, 8.0)	14.1 (10.0, 20.0)	56.1 (45.0, 70.0)	36.7 (32.0, 42.0)	26.4 ± 5.5
3 Months Postoperative	1.3 (1.0, 2.0)	6.3 (5.0, 10.0)	121.1 (115.0, 128.8)	73.4 (68.0, 73.5)	61.3 ± 8.1
Final Follow-up	0.5 (0.0, 1.0)	2.2 (0.0, 5.0)	137.7 (130.0, 145.0)	91.5 (89.3, 94.0)	83.8 ± 3.7

### 4.4 Clinical applications in neurosurgery

The application of tension-relieving suturing in neurosurgery includes procedures such as skull and spinal surgeries, including dural closure, tumor resection, and spinal fusion. By minimizing tissue deformation and cerebrospinal fluid leakage, tension-relieving suturing enhances surgical precision and reduces the risk of postoperative complications, such as meningitis and cerebrospinal fluid leaks. In an experimental study on median nerve suturing in mice (36), it was found that moderate tension had a positive effect on nerve recovery. Excessive tension can lead to nerve tissue damage and dysfunction, while reduced tension promotes nerve regeneration, shortens recovery time, and improves functional recovery rates. The experiment was divided into three groups: alleviated tension sutures (ATS), sutures under tension (SUT), and the control group, which did not undergo nerve suturing. The skin was incised, the median nerve was located and observed, and finally, the skin was sutured. The functional evaluation of the median nerve in mice was conducted through the time required for the first recovery of finger flexion, grip strength, and the quantitative measurement of atrophy in the flexor and pronator muscles. The results showed that the average time for finger flexion recovery in the ATS group was 13.7 days, while the SUT group showed no recovery. In the grip test, 180 days after surgery, the median nerve function in the ATS group had recovered to an average of 90.0% ± 4.5%. In contrast, after 180 days of observation, no evidence of median nerve function regeneration was found in the SUT group (as shown in Figure 8). In muscle mass evaluation, the muscle mass of the ATS group was significantly higher than that of the control and SUT groups, measuring 761 mg, while the latter was only 526 mg. This study suggests that in nerve suturing, relieving the tension at the suture site can maintain the stability of the nerve stump, promote axonal growth, and facilitate functional recovery, thereby accelerating the nerve healing process.

### 4.5 Clinical applications in cardiovascular surgery

Vascular anastomosis is a key technique in cardiovascular surgery, and the patency of the anastomosis is a critical indicator of postoperative success. Tension, as an important factor affecting the mechanical and biological properties of the anastomosis, has become a research hotspot. One of the fundamental principles of microvascular surgery is that there should not be excessive tension at the anastomosis site (37). As early as 1982, Chow et al. (38) reported that when arterial defects were sutured under tension, cracks would appear on the lumen surface. Researchers performed microvascular anastomosis on the femoral arteries of rats after excising blood vessels of varying lengths (distance between two branches). The study found that the longer the excised length, the higher the vessel patency rate, suture site permeability, and post-anastomosis vessel stenosis rate (see Table 7). Geng Menglu et al. (39) proposed a small vessel tension-relieving anastomosis technique. By comparing the changes in the vascular anastomosis under tension suturing and tension-relieving suturing, they found that when the tension was between 10° and 150°, leakage and tearing occurred at the anastomosis. After using tension-relieving suturing, although tension still existed between the injured vessels, the tension at the anastomosis site was reduced.

**Table 7:** Vessel patency rate, suture site permeability, and vessel stenosis rate after vascular anastomosis.

Excision length (%)	Patency rate (%)	Suture site permeability (%)	Vessel stenosis rate (%)
40	92	33	16
60	75	100	26
80	67	100	52

## 5 Limitations and poor prognosis of tension-relieving surgery

Although tension-relieving surgery is widely adopted and has achieved good results, it also faces potential complications and adverse events. As with any medical procedure, the safety of sutures must be carefully evaluated and monitored to ensure optimal patient care and minimize associated risks. In an analysis of adverse event reports related to medical suturing devices (40), which examined 409 cases of medical suture-related adverse events, it was found that the clinical manifestations of these adverse events were primarily suture reactions and poor wound healing (see Table 8). The severity of these adverse events, which require both internal and external surgical treatment to avoid permanent damage, accounted for 80.20% of the total reports. These complications may also arise from technical errors during the suturing process, inherent limitations of suturing materials, or patient-specific factors such as tissue fragility and immune responses (41).

**Table 8:** Analysis of Adverse Event Causes. Reproduced with permission from Chinese Journal of Pharmacovigilance (40).

Adverse event name	Number of cases	Manifestation	Possible causes
Breakage	27	Suture or needle breakage during suturing, separation of needle and thread; breakage of suture during healing period	Physical properties, absorption properties, model selection, suturing method
Suture Reaction	213	Redness, small pustules, or exudate, with no induration or tenderness of surrounding skin	Suture material, model selection, suturing method
Slow Absorption or Non-Absorption	39	Suture does not absorb within the specified period, some accompanied by incision redness, induration, exudate, suture reaction	Suture material, patient's constitution
Wound Infection	35	Redness, swelling, tenderness, purulent discharge in severe cases, some accompanied by systemic symptoms like fever	Sterilization failure, contamination during transport or use, surgical environment, aseptic technique, patient's constitution
Poor Wound Healing	95	Poor or slow healing of the incision, often accompanied by redness, induration, hematoma, fluid accumulation, and suppuration	Surgical trauma, rejection reaction, infection, patient constitution, suturing technique

Tension-relieving surgery may be associated with more severe complications, such as wound infection, foreign body reactions, or suture granuloma formation (42, 43). These adverse events often require timely identification and intervention, including suture removal, wound debridement, and antimicrobial treatment. Surgeons should closely monitor patients and strictly adhere to standardized surgical techniques during the procedure, continuing to observe for any suture-related complications during postoperative follow-up. Collaboration with an interdisciplinary team is essential to reduce the risk of adverse events and improve patient outcomes.

Although tension-relieving surgery has a high safety profile, poor prognosis may still occur in certain cases. This is mainly related to individual differences, surgical skills, and postoperative care. First, the effectiveness of tension-relieving surgery largely depends on the patient's individual physiological conditions, such as skin elasticity, age, wound location, and overall physical condition (29). Second, for thin and fragile skin, there is a risk of sutures protruding to the surface of the epidermis after suturing, and sutures left in the skin may increase the risk of infection (11).

When performing tension-relieving suturing, surgeons need to consider multiple factors to ensure the success of the surgery and postoperative recovery. These factors include the distance of the suture from the anastomosis edge, the relationship between suture spacing and edge distance, the choice of suture layers, tension control of the sutures, and the design of the anastomosis shape. Suture selection is essential for optimal wound healing and aesthetic outcomes. Based on absorbability, sutures are classified as absorbable (e.g., polyglactin 910, polyglycolic acid) for internal tissue closure, and non-absorbable (e.g., nylon, polypropylene) for skin closure requiring later removal. Sutures are also categorized by origin: natural sutures (e.g., catgut, silk) are easy to handle but may trigger stronger inflammatory responses, while synthetic sutures offer more predictable degradation and lower tissue reactivity. Structurally, monofilament sutures reduce infection risk due to their smooth surface, while multifilament sutures provide better knot security but may increase infection susceptibility (44). In addition to these factors, suture diameter also plays a critical role: studies have shown that large-diameter sutures tend to cause tissue tearing under high tension, while small-diameter sutures are more likely to break (45). Therefore, choosing the right suture depends on the wound location, tension requirements, infection risk, cosmetic goals, and mechanical demands during healing. However, despite these well-established criteria, surgeons may still rely more on their experience and intuition rather than objective evidence (46). This subjective tendency also extends to postoperative scar assessment. When evaluating patient scar characteristics, the scar scales used are relatively objective parameters. Although they have been widely adopted, human bias may still influence their application, and a fully objective method for scar evaluation has yet to be developed (28). Therefore, future research should focus on optimizing assessment tools and minimizing human interference to provide more accurate and reliable scar evaluation results.

## 6 Clinical tension measurement methods

**Figure 9:**
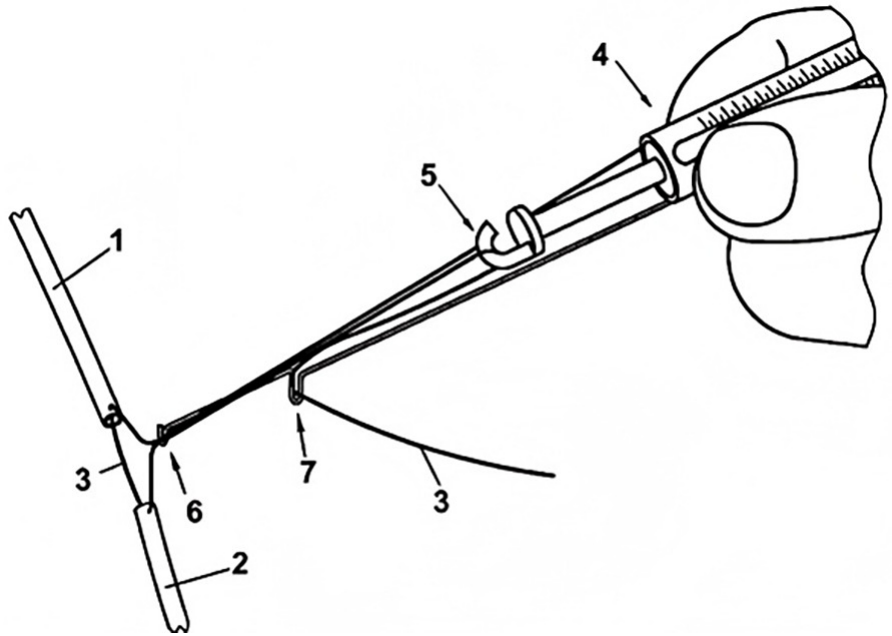
Tension Spring Balance. Reproduced with permission from J Plast Reconstr Aesthet Surg (53).

**Figure 10:**
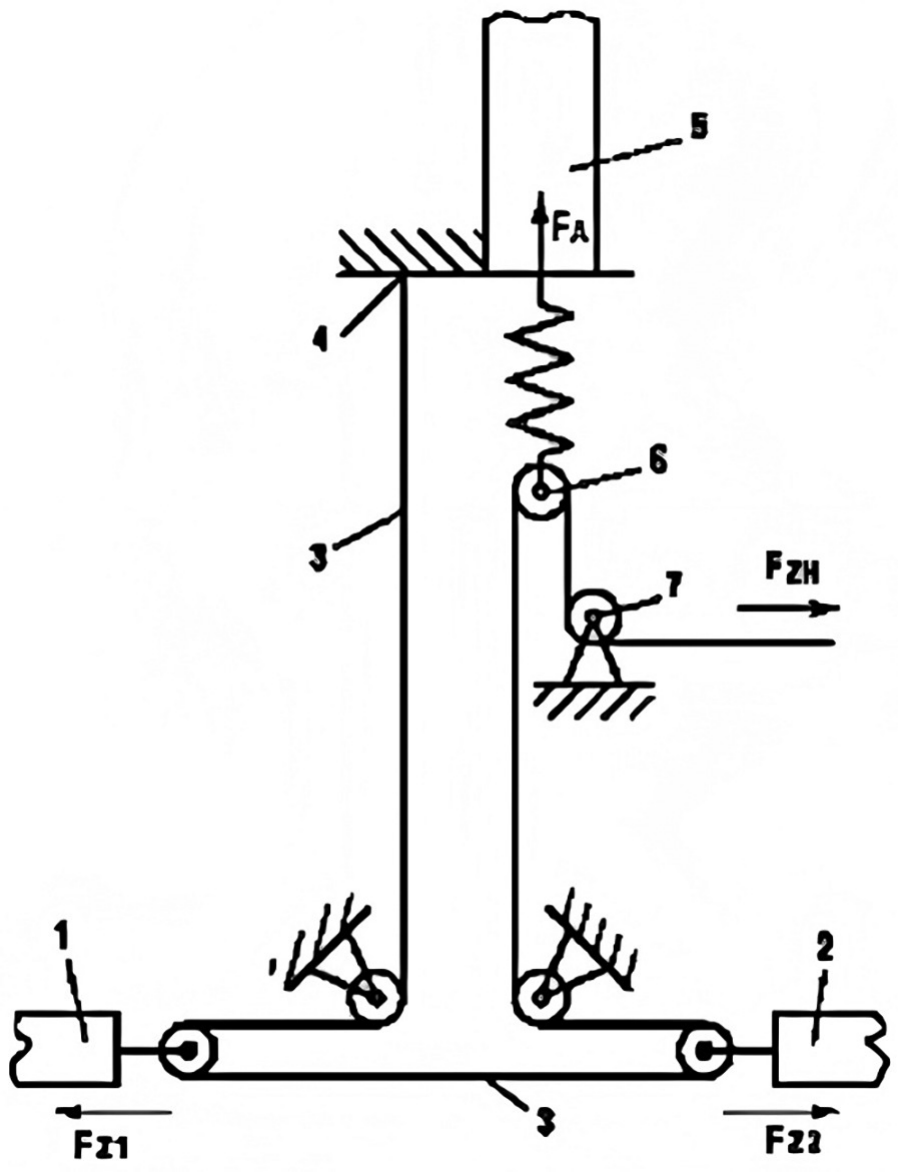
Tyrolean Tensiometer in Use. Reproduced with permission from J Plast Reconstr Aesthet Surg (53).

In microvascular repair surgery, the repair of dissected arteries sometimes must be performed under longitudinal tension. While current methods or devices can measure soft tissue tension values (47–51), there is no objective standard to determine whether the tension caused by direct suturing is within an acceptable range or whether tissue transplantation is required. Instead, reference values for tension in flap suturing after skin suturing with different tensions are explored (52). In a study involving tension measurement during vascular anastomosis (53), the design and validation of the Tyrolean tensiometer were described. The Tyrolean tensiometer was designed and validated to enable real-time assessment of vascular anastomotic tension. It employs a high-stiffness orthodontic wire spring (Figure 9) and demonstrates good measurement accuracy and ease of use. The device works by threading the suture through a U-shaped hook attached to the vessel ends. During traction, the spring scale indicates the amount of tension applied (Figure 10), helping surgeons determine if additional tension-relief measures are needed to improve surgical outcomes and long-term results. In practice, the suture's non-needle and needle ends are fixed to the vascular stumps. Both ends pass through the U-shaped hook; the needle end is connected to the spring hook and looped through a curved guide before being pulled to approximate the vessel ends. The resulting tension is displayed on the scale. After measurement, the suture is released from the hooks and tied as usual. For accuracy, the U-hook must be positioned directly above the intended anastomotic line.

To ensure accurate measurement, the U-shaped hook must be placed directly at the estimated anastomosis site. During measurement, the suture is passed through the two vessel stumps 1 and 2 as usual. Then, the needleless end of the suture is pressed against the tension spring scale 4 with a finger, and both ends of the suture are placed into the U-shaped hook 6. Next, the needle end of the suture is passed through the hook 5 of the tension spring scale and looped around the curved hook 7 to ensure longitudinal strain movement, then pulled tight. In this way, the two vessel stumps are brought together, and the applied tension can be read from the scale on the tension spring scale. After measurement, the suture is released from all hooks, and the first knot is tied as usual.

## 7 The application and prospects of mathematical methods and artificial intelligence in tension-relieving suture surgery

Mathematical methods and Artificial Intelligence (AI) especially finite element analysis and machine learning, provide revolutionary support for the planning and execution of tension-reducing surgeries like vascular anastomosis. However, it involves long surgical times, high technical requirements, and may lead to a range of complications (54). Therefore, many researchers have sought methods to ensure anastomosis quality by adjusting the appropriate values of process variables (55–60). The Smart Tissue Autonomous Robot (STAR), an autonomous robotic surgical intervention system designed by Axel Kriger, has demonstrated the ability to match human surgeons in intestinal anastomosis (55). After the surgeon approves the surgical plan, the system can autonomously perform the surgery. STAR creates a suturing plan by evaluating tissue thickness and structure, and autonomously completes the suturing after confirmation by the surgeon (61). Additionally, the system maintains continuous communication with the surgeon to adapt to intraoperative tissue deformation or unexpected changes (61).

Moreover, finite element models of human skin can be applied to interactive real-time surgical simulations, such as using skin flaps for facial reconstruction. Excessive tissue tension reduces blood supply and leads to tissue necrosis. Tepole et al. (62) used finite element methods to simulate stress distribution in skin flaps during reconstructive surgery, and the results showed that the maximum stress area in the flap closely matched the area of tissue necrosis. Using real-time finite element models to simulate the stretching process of skin flaps helps guide the surgeon in precise stretching, avoiding over-stretching or insufficient stretching, thereby improving surgical accuracy and safety (63). The use of skin substitutes is a treatment for third-degree burns, but the skin substitutes used need to have mechanical properties similar to human skin (64). Yu et al. (65) used finite element modeling and 3D printing technology to design and analyze skin substitutes with anisotropic mechanical properties. The study performed simulation calculations for Young's modulus and verified its rationality through uniaxial tensile experiments, finding that different structural designs can regulate the mechanical properties of skin substitutes to make them closer to natural skin.

The application of AI in robotic surgery enhances precision, efficiency, and accessibility through functions like image recognition, motion control, and tactile feedback. The advantages of AI integration include increased precision, reduced surgeon fatigue, and enhanced safety (65). Zhu et al. (66) developed a robot for oral surgery, embedding piezoelectric sensors at the instrument tips capable of detecting tissue forces up to 15 N. These sensed forces can be displayed to the surgeon on a console, helping them perceive changes in resistance during tissue separation and suturing to avoid excessive traction force. Mohammed et al. (67) used a convolutional neural network model, Xception, to evaluate the success rate of surgical suturing with an accuracy rate of 95%. These technologies not only provide powerful tools for surgeons but also offer safer and more efficient surgical treatment options for patients. Li et al. (68) employed digital image correlation to quantitatively analyze the tension distribution along the wound edges during flap suturing, demonstrating the effectiveness of tension-relieving suturing in enhancing flap perfusion, reducing local stress, and promoting healing. In contrast, Cai et al. (69) utilized numerical simulations to investigate the effects of mechanical tension on wound remodeling and scar formation, revealing that appropriate tension regulation can optimize cellular signaling and local microcirculation, thereby facilitating wound healing and reducing the incidence of abnormal scarring. Moreover, Yang (70) applied tension-relieving technology in anterior cruciate ligament reconstruction, with biomechanical analyses indicating that the internal tension-relieving technique not only enhanced the stability of the reconstructed ligament but also improved patients' functional recovery. Taken together, these studies—ranging from quantitative measurements and numerical simulations to clinical applications—systematically elucidate the critical role of tension-relieving techniques in reducing surgical tension, optimizing wound repair, and enhancing functional outcomes, thereby providing a theoretical basis and practical support for further optimization of surgical procedures.

The continuous improvement and innovation of tension-relieving suturing techniques have brought great hope for advancing surgical skills and improving patient care. In the future, the application of preoperative modeling and simulation technologies may further optimize surgical plans, improve surgical precision, reduce surgical risks, and open up broader prospects for the development of tension-relieving suture surgery. Combined with finite element simulation technology, it can simulate suturing surgeries under tension, studying the interaction between sutures and surrounding tissues (71). It can also analyze the stress distribution patterns of different excision shapes (72). Additionally, it can be used to analyze the impact of different suture materials on tissue stress distribution, thereby optimizing the performance of suture materials (73). This forward-looking analysis can provide valuable decision support for doctors, ensuring the safety and effectiveness of surgeries.

## 8 Conclusion

As a significant innovation in the field of surgery, tension-relieving suture techniques have effectively alleviated intraoperative mechanical stress by optimizing suture materials and design. These techniques have markedly reduced the incidence of wound dehiscence, scarring, and soft tissue complications, demonstrating particular efficacy in plastic, neurosurgical, and cardiovascular procedures. Clinical evidence indicates that such techniques not only enhance surgical success rates and patient satisfaction but also achieve a balance between functional outcomes and aesthetic appearance by precisely controlling wound approximation and tissue stress. However, challenges remain. Suture breakage, knot slippage, and tissue reactions may result from limitations in suture materials, operative techniques, or individual patient variability. These issues highlight the need for standardized intraoperative practices and personalized postoperative surveillance. Future breakthroughs in tension-relieving techniques are expected to focus on three major directions: (1) Material innovation, aiming to develop intelligent sutures with enhanced biocompatibility, antibacterial activity, and biodegradability—such as nano-coated or absorbable composite materials—to minimize infection risk and optimize the healing process; (2) Technological integration, where artificial intelligence and 3D printing will drive the customization of suture strategies, such as predicting tension distribution based on imaging data for preoperative planning, and integrating multimodal data (e.g., genomics, metabolic indicators) to build more accurate models for postoperative complication prediction; (3) Cost control, which remains a major constraint, requiring policy support and industrial scaling to reduce the threshold for high-tech applications and ensure equitable access.

Moreover, our institution is actively developing a multicenter plastic surgery database. Due to varying surgical techniques and levels of experience among surgeons, establishing such a database is crucial for aggregating treatment strategies from clinicians across different regions, including patients' long-term follow-up results. By comparing these follow-up outcomes, it becomes possible to identify which surgical method is more effective for a given condition. This continuous feedback loop not only facilitates the ongoing refinement of surgical techniques but also paves the way for establishing a gold standard approach.

The authors suggest that the advancement of tension-relieving suture techniques marks a paradigm shift in surgical practice—from experience-based to data-driven approaches. Nevertheless, their full potential has yet to be realized. Current AI models often rely on single-source data; future developments should emphasize the integration of multidimensional information to achieve truly precise treatment. Additionally, the long-term biocompatibility and environmental sustainability of suture materials require further investigation.

In the future, with continued progress in smart materials, robotic-assisted surgery, and interdisciplinary collaboration, tension-relieving techniques are expected to become standardized tools in complex surgeries. This evolution will propel surgical practice toward the goals of “zero tension and zero complications,” ultimately improving patient outcomes and the overall efficiency of healthcare systems.
