# Correction: Progress in tension-relieving suturing surgery: revolutionary surgical techniques and patient prognosis evaluation methods

**DOI:** 10.3389/fsurg.2025.1641832

**Published:** 2025-06-25

**Authors:** Maolong Ge, Weifeng Zheng, Ping Yao, Lin Gao, Xinyang Ge, Qixin Zhang, Xiaolin Wang, Chuangju Guo

**Affiliations:** ^1^ College of Mathematical Medicine, Zhejiang Normal University, Jinhua, China; ^2^ Department of Cosmetic Dermatology, Hangzhou Plastic Surgery Hospital, Hangzhou, Zhejiang, China; ^3^ Department of Plastic Surgery, Hangzhou Plastic Surgery Hospital, Hangzhou, Zhejiang, China; ^4^ Research and Development Department, Zhejiang Demetics Medical Technology Co., Ltd., Hangzhou, China

**Keywords:** tension-relieving suture surgery, surgical innovation, tension-related complications, wound healing, surgical prognosis

A Correction on Progress in tension-relieving suturing surgery: revolutionary surgical techniques and patient prognosis evaluation methods By Ge M, Zheng W, Yao P, Gao L, Ge X, Zhang Q, Wang X and Guo C (2025). Front. Surg. 12:1587582. doi: 10.3389/fsurg.2025.1587582

In the published article, there were errors in the captions for Figure 1, Figure 2, Figure 3, Figure 4, Figure 5, Figure 6, Figure 7, Figure 8, Figure 9, Figure 10, Table 3, Table 4, Table 5, Table 6, and Table 8. The captions failed to include the original sources. There was also an error in the department for affiliation 2. The corrected captions and affiliation appear below.

For Figure 1, instead of ‘*(A) Buried dermal suture: before suturing (left), after suturing (right) (16). (B) Vertical mattress suture and its modified techniques: (a) BVMS. (b) MBVMS. (c) WEMVMS. (d) SBDS (17)’*, the corrected caption should be ‘*(A) Buried dermal suture: before suturing (left), after suturing (right). (B) Vertical mattress suture and its modified techniques: (a) BVMS. (b) MBVMS. (c) WEMVMS. (d) SBDS. Reproduced with permission from Acta Derm Venereol (16) and Chinese Journal of Reparative and Reconstructive Surgery (17)’*.

For Figure 2, instead of ‘*(A) Continuous horizontal mattress inversion suture. Buried horizontal mattress suture: (B) PBHMS. (C) FBHMS’,* the corrected caption should be ‘*(A) Continuous horizontal mattress inversion suture. Buried horizontal mattress suture: (B) PBHMS. (C) FBHMS. Reproduced with permission from Chinese Journal of Reparative and Reconstructive Surgery (17)’*.

For Figure 3, instead of ’*Schematic diagram of the modified FBHMS technique. (A) Suture path passing through one side of the incision; (B) suture path passing through both sides of the incision; (C) diagram after knotting (21)’,* the corrected caption should be ’*Schematic diagram of the modified FBHMS technique. A. Suture path passing through one side of the incision; B. Suture path passing through both sides of the incision; C. Diagram after knotting. Reproduced with permission from Ann Plast Surg (21)’*.

For Figure 4, instead of ‘*Control group: FBHMS experiment (A, B); experimental group: modified FBHMS experiment (C, D) (21). After 100 cycles of periodic mechanical stress, the incision gap in the control group widened further, while the gap in the experimental group remained narrow’*, the corrected caption should be ‘*Control group: FBHMS experiment (A, B); Experimental group: Modified FBHMS experiment (C, D). After 100 cycles of periodic mechanical stress, the incision gap in the control group widened further, while the gap in the experimental group remained narrow. Reproduced with permission from Ann Plast Surg (21)’*.

For Figure 5, instead of ‘*Representative clinical photographs of the first group (top): A 24-year-old female with a facial scar, treated with W-plasty incision suturing and CTR therapy. (A) Preoperative; (B) 12 months postoperatively; Preoperative VSS score: 5, postoperative VSS score: 1; Preoperative VAS score: 60, postoperative VAS score: 95. Representative clinical photographs of the second group (bottom): (A) 14-year-old female with a facial scar, treated only with W-plasty incision suturing. (A) Preoperative; (B) On the day of surgery; (C) One week postoperatively (after suture removal); (D) 12 months postoperatively; Preoperative VSS score: 7, postoperative VSS score: 4; Preoperative VAS score: 40, postoperative VAS score: 75 (27)’*, the corrected caption should be ‘*Representative clinical photographs of the first group (top): A 24-year-old female with a facial scar, treated with W-plasty incision suturing and CTR therapy. A. Preoperative; B. 12 months postoperatively; Preoperative VSS score: 5, postoperative VSS score: 1; Preoperative VAS score: 60, postoperative VAS score: 95. Representative clinical photographs of the second group (bottom): A 14- year-old female with a facial scar, treated only with W-plasty incision suturing. A. Preoperative; B. On the day of surgery; C. One week postoperatively (after suture removal); D. 12 months postoperatively; Preoperative VSS score: 7, postoperative VSS score: 4; Preoperative VAS score: 40, postoperative VAS score: 75. Reproduced with permission from Int Wound J (27)’*.

For Figure 6, instead of ‘*(A) Tension-relieving suturing allows new blood vessels to pass through the sutures around the anastomosis. (B) Moderate suture tension causes vessels to displace along the anastomosis. (C) High suture tension creates an avascular zone with vessel discoloration (34)’*, the corrected caption should be ‘*(A) Tension-relieving suturing allows new blood vessels to pass through the sutures around the anastomosis. (B) Moderate suture tension causes vessels to displace along the anastomosis. (C) High suture tension creates an avascular zone with vessel discoloration. Reproduced with permission from Am J Surg (34)’*.

For Figure 7, instead of ‘*The doctor uses sutures, plates, and nails to fix the fracture site (left); (left-A) suture hook passage; (left-B) suture retrieval; (left-C) loop formation; (left-D) tibial tunnel creation; (left-E) button plate & guide-wire setup; (left-F) final tensioning & anchor fixation; CT scan of a 28-year-old female patient with a right posterior cruciate ligament avulsion fracture figure (right). (right-A), x-ray of the fracture; (right-B), x-ray taken on the first day after surgery; (right-C), CT image three months after surgery; (right-D), bone fracture healing (35)’,* the corrected caption should be ‘*The doctor uses sutures, plates, and nails to fix the fracture site (left); CT scan of a 28- year-old female patient with a right posterior cruciate ligament avulsion fracture figure (right). A, x-ray of the fracture; B, x-ray taken on the first day after surgery; C, CT image three months after surgery; D, Bone fracture healing. Reproduced with permission from J Orthop Surg Res (35)’*.

For Figure 8, instead of ‘*The effect of different surgical methods on the grip ability of wistar rats. Results range from 30 days to 180 days. (A) Alleviated tension suture group. (B) Suture under tension group; Control group c did not undergo surgery, with each group representing the average score of 10 animals. Symbols indicate differences between groups (36)’,* the corrected caption should be ‘*The effect of different surgical methods on the grip ability of Wistar rats. Results range from 30 days to 180 days. (A) Alleviated tension suture group. (B) Suture under tension group; Control group (C) did not undergo surgery, with each group representing the average score of 10 animals. Symbols indicate differences between groups. Reproduced with permission from Microsurgery (36)’*.

For Figure 9, instead of ‘*Tension spring balance’,* the corrected caption should be ‘*Tension Spring Balance. Reproduced with permission from J Plast Reconstr Aesthet Surg (53)’*.

For Figure 10, instead of ‘*Tyrolean tensiometer in use’,* the corrected caption should be ‘*Tyrolean Tensiometer in Use. Reproduced with permission from J Plast Reconstr Aesthet Surg (53)’*.

For Table 3, instead of ’*Statistical data on patients and facial scars’*, the corrected caption should be ’*Statistical data on patients and facial scars. Reproduced with permission from Int Wound J (27)’*.

For Table 4, instead of ‘*Patient demographics and preoperative and postoperative scar assessment parameters’,* the corrected caption should be ‘*Patient demographics and preoperative and postoperative scar assessment parameters. Reproduced with permission from BMC Surgery (28)’*.

For Table 5, instead of ‘*Preoperative and postoperative scar width and assessment parameters’,* the corrected caption should be ‘*Preoperative and postoperative scar width and assessment parameters. Reproduced with permission from Journal of Cosmetic Dermatology (29)’*.

For Table 6, instead of ‘*Comparison of pain, knee range of motion, and function before and after surgery in patients’,* the corrected caption should be ‘*Comparison of pain, knee range of motion, and function before and after surgery in patients. Reproduced with permission from J Orthop Surg Res (35)’*.

For Table 8, instead of ‘*Analysis of adverse event causes’,* the corrected caption should be ‘*Analysis of Adverse Event Causes. Reproduced with permission from Chinese Journal of Pharmacovigilance (40)’*.

For affiliation 2, instead of *Department of Aesthetic Dermatology, Hangzhou Plastic Surgery Hospital, Hangzhou, Zhejiang, China*, the corrected affiliation should be *Department of Cosmetic Dermatology, Hangzhou Plastic Surgery Hospital, Hangzhou, Zhejiang, China*.

The authors apologize for these errors and state that this does not change the scientific conclusions of the article in any way. The original article has been updated.

